# Cerebral Autoregulation Monitoring to Evaluate for Clinical Outcome After Decompressive Hemicraniectomy for Acute Ischemic Stroke: Case Series

**DOI:** 10.3390/reports9020095

**Published:** 2026-03-24

**Authors:** Julia E. Alexander, Daniel R. Felbaum, Jeffrey C. Mai, Jason J. Chang

**Affiliations:** 1 School of Medicine, Georgetown University, Washington, DC 20007, USA; jea116@georgetown.edu; 2 Department of Neurosurgery, Georgetown University and MedStar Washington Hospital Center, Washington, DC 20007, USA; 3 Department of Critical Care Medicine, MedStar Washington Hospital Center, Washington, DC 20010, USA; 4 Department of Neurology, Georgetown University Medical Center, Washington, DC 20007, USA

**Keywords:** decompressive hemicraniectomy, ischemic stroke, intracranial pressure, pressure reactivity index, cerebral autoregulation

## Abstract

**Background and Clinical Significance**: Decompressive hemicraniectomy (DHC) is a life-saving intervention for malignant middle cerebral artery (MCA) infarction, but postoperative secondary injury mechanisms and functional outcome remain difficult to evaluate using intracranial pressure (ICP) alone. The pressure reactivity index (PRx), calculated as the moving correlation coefficient between ICP and mean arterial pressure (MAP), provides a measure of cerebral autoregulation. The utility of PRx monitoring in ischemic stroke, especially following DHC, remains uncertain. **Case Presentation**: We describe two patients presenting with acute ischemic stroke in the MCA territory who underwent DHC followed by postoperative ICP and PRx monitoring. Case 1 is a 40-year-old female with a left proximal MCA occlusion initially treated with endovascular thrombectomy (EVT) who required emergent DHC due to re-occlusion. Postoperatively, ICPs remained controlled, and PRx values were favorable (<0.2), indicating preserved cerebral autoregulation. She later showed moderate neurological improvement. Case 2 was a 68-year-old female with a left proximal MCA occlusion treated with EVT who developed worsening cerebral edema and midline shift, necessitating emergent DHC. Despite adequate ICP control, PRx values remained markedly elevated (0.45 to 0.73), consistent with impaired cerebral autoregulation, and her neurologic state remained poor at discharge. **Conclusions**: These contrasting cases suggest that PRx may provide physiologic information not reflected by ICP metrics alone post-DHC. PRx monitoring may provide complementary physiologic insight into postoperative autoregulatory status following DHC. Further investigation is warranted to define its role in individualized post-DHC management and prognostication in malignant ischemic stroke.

## 1. Introduction and Clinical Significance

Malignant middle cerebral artery (MCA) infarction leads to rapid cerebral edema, increased intracranial pressure (ICP), decreased cerebral perfusion pressure (CPP), and high mortality if untreated [1,2]. Decompressive hemicraniectomy (DHC) is an established surgical intervention to control ICP and prevent herniation. Randomized trials—including DECIMAL, DESTINY, HAMLET, and DESTINY-II—demonstrated that DHC reliably reduces mortality in malignant MCA infarction [1,2,3,4]. However, these trials consistently showed limited improvement in long-term functional outcome, demonstrating that ICP control alone does not determine neurological recovery.

This disconnect between ICP control and functional outcome is not unique to ischemic stroke. The landmark BEST:TRIP trial in traumatic brain injury found that ICP-targeted therapy did not improve functional outcome compared with management based on clinical exam and imaging, indicating that ICP is an important but incomplete surrogate for underlying cerebral physiology [5]. Given that DHC disrupts the closed cranial vault and alters intracranial compliance, traditional ICP metrics may become less reflective of underlying cerebral physiology. Therefore, additional physiologic markers beyond ICP are needed to assess residual cerebral viability after DHC.

The pressure reactivity index (PRx) quantifies cerebrovascular autoregulation as a moving correlation between slow waves of ICP and mean arterial pressure (MAP) [6,7]. Negative or near-zero PRx values indicate intact autoregulation, while positive values suggest impaired reactivity. PRx has been extensively studied in severe traumatic brain injury, where deviations from the optimal CPP correlated with poor outcome [6]. The COGiTATE trial demonstrated the feasibility of individualized CPP management based on continuous PRx-derived targets [8]. Additionally, prior work in acute brain injury populations undergoing craniectomy suggests that PRx may remain measurable and retain physiologic signal characteristics despite disruption of the cranial vault [9,10]. However, PRx accuracy and prognostic significance in mass lesion pathologies such as large ischemic stroke, particularly following decompressive surgery, remain unclear.

This case series is clinically significant because it describes two patients with malignant MCA infarction who underwent DHC with postoperative PRx monitoring. It examines whether PRx dynamics continue to reflect cerebral autoregulatory capacity and correlate with functional outcomes despite adequate ICP control following surgical decompression.

## 2. Case Presentation

### 2.1. Neuromonitoring and PRx Analysis

Following DHC, ICP and MAP values were recorded at one-minute intervals using bedside neuromonitoring systems with arterial line and intraparenchymal bolt placement (Neurovent-PTO, Raumedic, Mills River, NC, USA). Data was acquired and processed using the CNS Envision platform (Natus, Ambler, PA, USA). CPP was calculated as MAP minus ICP. PRx was generated as a moving Pearson correlation coefficient between ICP and MAP over sequential time windows as noted prior [11]. For Case 1, monitoring occurred for approximately 38 h. For Case 2, monitoring occurred for approximately 69 h. Daily mean PRx values were calculated by averaging all available PRx measurements within each monitoring interval. Data segments with signal dropout or artifact were excluded according to standard bedside waveform quality controls. No additional post hoc signal manipulation was performed.

### 2.2. Case 1

**Figure 1:**
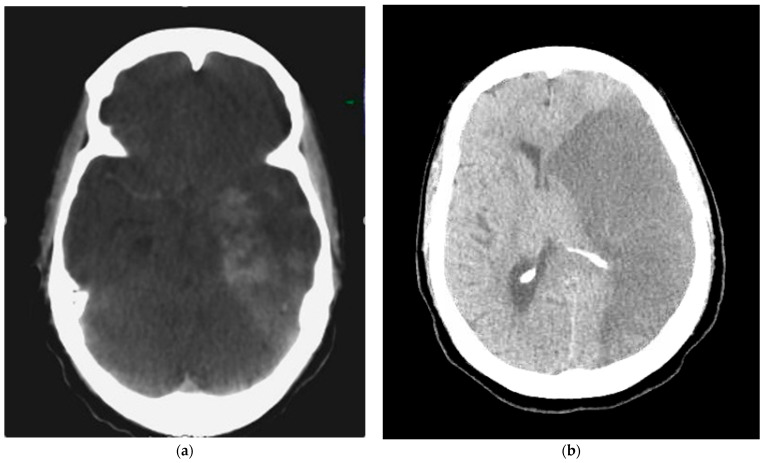
Initial imaging for both cases showing large middle cerebral artery (MCA) infarctions prior to decompression. (**a**) Case 1 with early malignant left MCA infarction and hemorrhagic transformation in the left MCA territory captured immediately after endovascular thrombectomy. (**b**) Case 2 with a complete and malignant left MCA infarction with 8 mm left to right midline shift and effacement of the left lateral ventricle.

**Figure 2:**
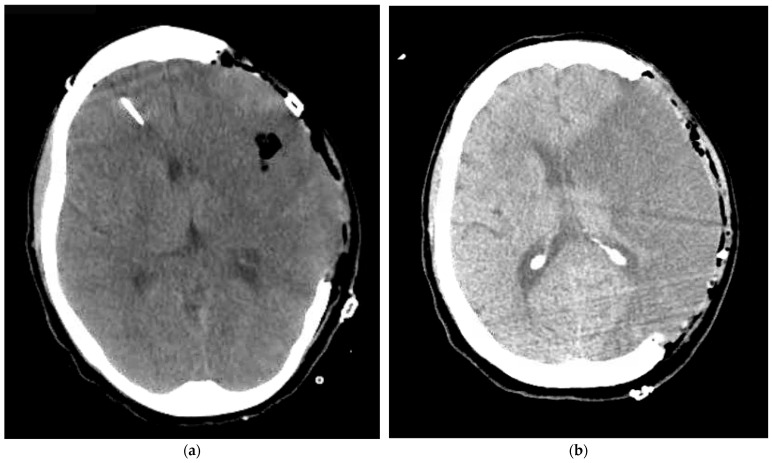
Postoperative imaging demonstrating improved midline shift, stable middle cerebral artery infarct evolution, and stable cerebral edema after decompressive hemicraniectomy in (**a**) Case 1 and (**b**) Case 2.

A 40-year-old female presented with an initial NIH Stroke Score (NIHSS) of 4 and was found to have a left MCA infarct with proximal left MCA occlusion. She was ineligible for intravenous Tenecteplase, but endovascular thrombectomy (EVT) was performed with a “thrombolysis in cerebral infarction” (TICI) grade 3 reperfusion. On hospital day 2, the patient was extubated with a Glasgow Coma Scale (GCS) of 15, but developed new-onset aphasia, right-sided facial droop, and right-sided flaccidity 1.5 h later. Repeat imaging showed occlusion of the left internal carotid artery at the carotid bifurcation and repeat EVT was performed with TICI 2a reperfusion. Following this, a dyna-computed tomography of the head showed a malignant left MCA stroke with hemorrhagic transformation (Figure 1a). Subsequent examination showed a decreased left pupillary response. An emergent left DHC was performed with stable infarct volume and improved midline shift (Figure 2a). Postoperatively, a right frontal bolt was placed for ICP monitoring. During hospital days 2–4, average daily PRx values remained below 0.2 (overall average PRx value 0.15) with an overall average ICP value of 13.44 mmHg and CPP value of 65.13 mmHg (Table 1). These findings were consistent with preserved cerebral autoregulation despite the initial pre-DHC herniation occurring. The ICP monitor was removed on hospital day 4, and she was extubated on hospital day 8. Serial computed tomography of the head (CTH) scans remained stable through hospital day 19. Her hospital course was characterized by a wound infection—treated with a washout and antibiotics. However, she recovered and was subsequently transferred to acute inpatient rehabilitation on hospital day 32. Upon discharge from rehabilitation 2 months later, she was awake but debilitated with a Modified Rankin Scale (mRS) of 4.

### 2.3. Case 2

A 68-year-old female presented with an NIHSS score of 15 due to a proximal left MCA occlusion. EVT was performed with TICI grade 3 reperfusion. However, she was noted to have underlying atherosclerotic disease with severe MCA stenosis, requiring placement of an acute intracranial stent (Neuroform Atlas 4.0 × 21 mm). Post-angioplasty, she was placed on an intravenous cangrelor drip and experienced no hemorrhage or ischemic progression on postoperative CTHs. However, interval CTH on hospital day 4 showed worsening malignant cerebral edema and increased 8 mm left-to-right midline shift (Figure 1b), prompting emergent left DHC followed by placement of a right frontal ICP bolt. Imaging after decompression showed stable infarct volume and improved midline shift (Figure 2b). Immediately following DHC, PRx values were markedly elevated at 0.73, indicating severely impaired cerebral autoregulation. Subsequent PRx values on hospital days 5–7 all remained impaired with a mean PRx of 0.56. Her ICPs remained well controlled throughout with an average value of 7.27 mmHg, and she had adequate CPP values with an average of 67.24 mmHg (Table 1). Her post-operative findings showed an adequate DHC with controlled ICPs, but persistently impaired cerebral autoregulation. Her clinical exam never improved, and she required a tracheostomy and percutaneous endoscopic gastrostomy on hospital day 9. On hospital day 19, she had a mRS of 5 and was transferred to a long-term acute care facility for ventilator weaning. Two months after her stroke, her clinical outcome remained poor and unchanged with a mRS of 5.

## 3. Discussion

DHC is highly effective at reducing ICP and preventing herniation in malignant MCA infarction. However, as demonstrated in malignant stroke trials evaluating DHC and reinforced by the BEST:TRIP trial in traumatic brain injury, normalization of ICP does not guarantee improved neurological recovery [5]. This discrepancy highlights that ICP control alone does not fully characterize the underlying cerebral physiology that determines outcome. Here we present two cases, where despite achieving ICP control with DHC, cerebral autoregulation values, represented by PRx, diverged widely and appeared to parallel clinical trajectory.

Together, these cases suggest that ICP alone is insufficient to characterize cerebral physiology after DHC. Although DHC disrupts the rigid cranial vault and improves intracranial compliance, it does not necessarily improve cerebral autoregulation. PRx is derived from the dynamic relationship between slow-wave fluctuations in ICP and MAP, reflecting active vasomotor responses rather than absolute pressure values alone. Therefore, cerebral autoregulation indices such as PRx may capture persistent autoregulatory failure and help with prognostication, even when ICPs are well-controlled.

In these two cases, both patients achieved good ICP control and improved, stable postoperative imaging due to their DHCs. However, patient 1 with preserved PRx values demonstrated neurological improvement. Conversely, patient 2 had persistently impaired PRx values and poor neurological recovery. These contrasting trajectories suggest that PRx may capture ongoing autoregulatory failure and secondary microvascular dysfunction that is not reflected by absolute ICP values.

Our two-patient case series has several important strengths and limitations. A key strength is the use of continuous multimodal neuromonitoring to directly compare ICP and cerebral autoregulation following DHC, allowing physiologic divergence to be demonstrated despite controlled ICP values. The inclusion of two patients with comparable surgical intervention but contrasting PRx values and outcomes further highlights the potential prognostic relevance of cerebral autoregulation monitoring. However, this report is limited by its small sample size and observational nature. In addition, PRx has been more extensively validated in traumatic brain injury than in ischemic stroke, and extrapolation across pathologies must be made cautiously. Potential confounders, including age, infarct burden, timing of decompression, and systemic complications, may also have influenced outcomes. Given the small sample size and observational design, these findings should be interpreted as hypothesis-generating and require validation in larger prospective cohorts.

The observations from these two cases support the hypothesis that PRx may provide complementary physiologic information beyond ICP of residual cerebral viability and potential for recovery following DHC. This observation may also apply to focal pathologies such as malignant ischemic stroke. Larger prospective studies are warranted to determine whether integration of PRx into post-DHC neuromonitoring can improve prognostication or guide individualized CPP-targeted therapy.

## 4. Conclusions

DHC reliably reduces ICP in malignant ischemic stroke, but effective ICP control alone and improved imaging does not ensure neurological recovery. In this two-patient case series, despite adequate ICP control in both patients, postoperative PRx trajectories differed markedly and were associated with diverging clinical outcomes. These findings suggest that post-DHC, PRx may provide complementary physiological information beyond ICP of residual cerebral viability and potential for recovery. Further prospective investigation is needed to define the role of PRx-guided monitoring and management following decompressive surgery in ischemic stroke.

## Figures and Tables

**Table 1:** Summary of average daily values of pressure reactivity index (PRx), intracranial pressure (ICP), cerebral perfusion pressure (CPP), and mean arterial pressure (MAP) for two patients with malignant middle cerebral artery strokes receiving decompressive hemicraniectomy. Patient 1 transferred to acute rehabilitation and Patient 2 was transferred to a ventilator weaning facility.

	Case 1				Case 2			
Hospital Day	PRx	Mean ICP (mmHg)	Mean CPP (mmHg)	Mean MAP (mmHg)	PRx	Mean ICP (mmHg)	Mean CPP (mmHg)	Mean MAP (mmHg)
**1**								
**2**	0.16	10.27	71.08	81.35				
**3**	0.13	13.79	66.26	81.17				
**4**	0.17	16.26	58.07	74.33	0.73	6.12	66.39	72.48
**5**					0.46	7.02	65.89	72.72
**6**					0.59	7.69	67.81	75.45
**7**					0.45	8.24	68.86	77.06
**Average Mean**	0.15	13.44	65.13	78.95	0.56	7.27	67.24	74.43

## Data Availability

The data supporting the findings of this study are not publicly available due to patient privacy but are available from the corresponding author upon reasonable request.

## Abbreviations

The following abbreviations are used in this manuscript:

MCA	Middle cerebral artery
ICP	Intracranial pressure
CPP	Cerebral perfusion pressure
DHC	Decompressive hemicraniectomy
PRx	Pressure reactivity index
MAP	Mean arterial pressure
EVT	Endovascular thrombectomy
NIHSS	NIH stroke score
TICI	Thrombolysis in cerebral infarction
GCS	Glasgow coma scale
CTH	Non-contrast computed tomography of the head
mRS	Modified Rankin scale
